# Supplementary material for: Thin Film Composite Membranes Based on the Polymer of Intrinsic Microporosity PIM-EA(Me2)-TB Blended with Matrimid®5218
Source: Membranes (Basel). 2022 Sep 13;12(9):881. doi: 10.3390/membranes12090881 (PMC9502825; doi:10.3390/membranes12090881)
Supplement: Supplementary file 1 [file membranes-12-00881-s001.zip › membranes-1865992-supplementary.pdf]

membranes

Supplementary Information

Thin film composite membranes based on the polymer of intrinsic microporosity PIM-EA(Me_2)-TB blended with Matrimid®5218.

Mariagiulia Longo[1], Marcello Monteleone[1], Elisa Esposito[1*], Alessio Fuoco[1], Elena Tocci[1], Maria-Chiara Ferrari[2], Bibiana Comesaña-Gándara[3], Richard Malpass-Evans[3], Neil B. McKeown[3], Johannes C. Jansen[1*]

[1]Institute on Membrane Technology (CNR-ITM), Via P. Bucci, 17/C, 87036 Rende (CS), Italy
[2] School of Engineering, University of Edinburgh, Robert Stevenson Road, Edinburgh EH9 3FB, UK
[3] EaStCHEM, School of Chemistry, University of Edinburgh, David Brewster Road, Edinburgh EH9 3FJ, UK
* Correspondence: e.esposito@itm.cnr.it (E.E.); johannescarolus.jansen@cnr.it (J.C.J.)

	Matrimid/PIM ratios (wt%)				
Support	0/100	25/75	50/50	75/25	100/0
Vladipor Fluoroplast 50 nm					
Sulzer PAN UF					
Synder PAN 30kDa					
Nanostone PVDF PV350					

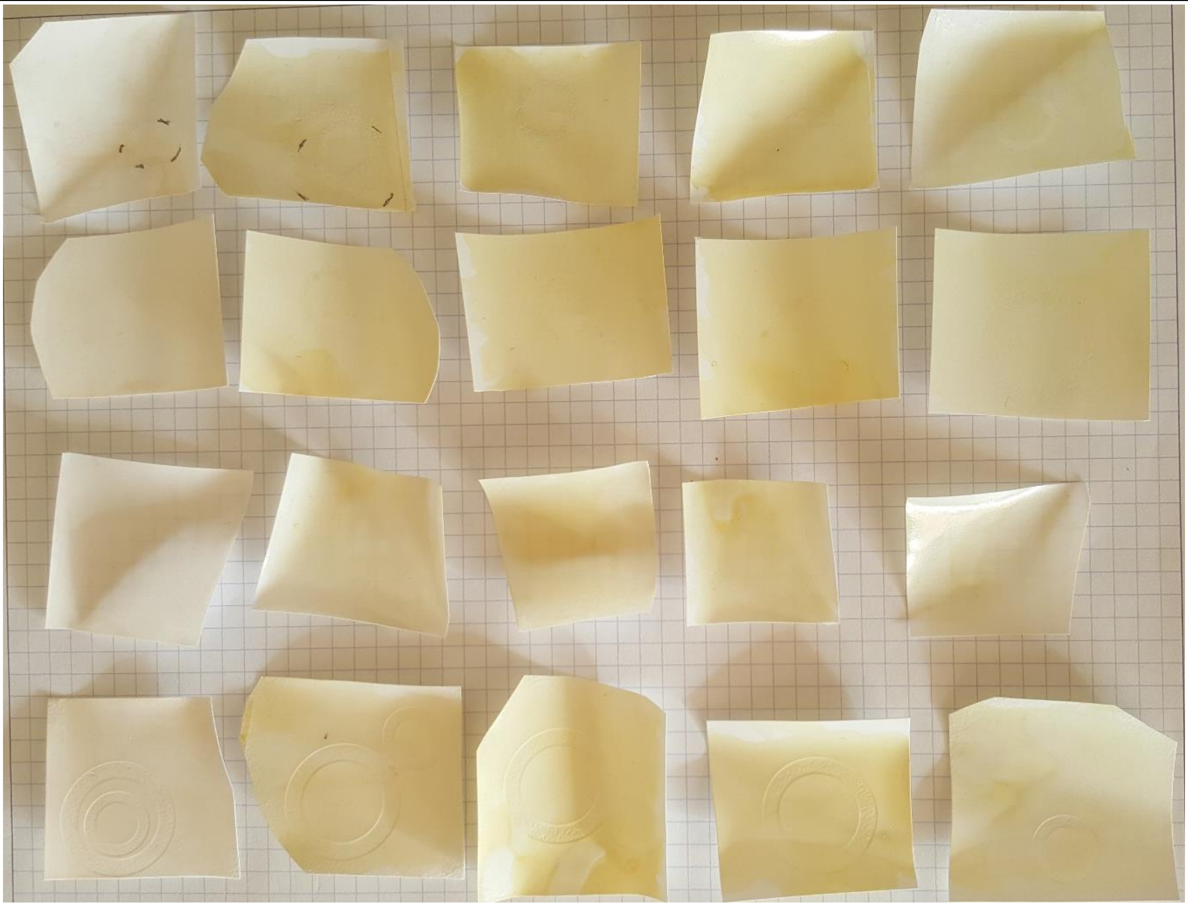

SI Figure S1. Images of the TFC membranes with the four different supports after the permeability tests.

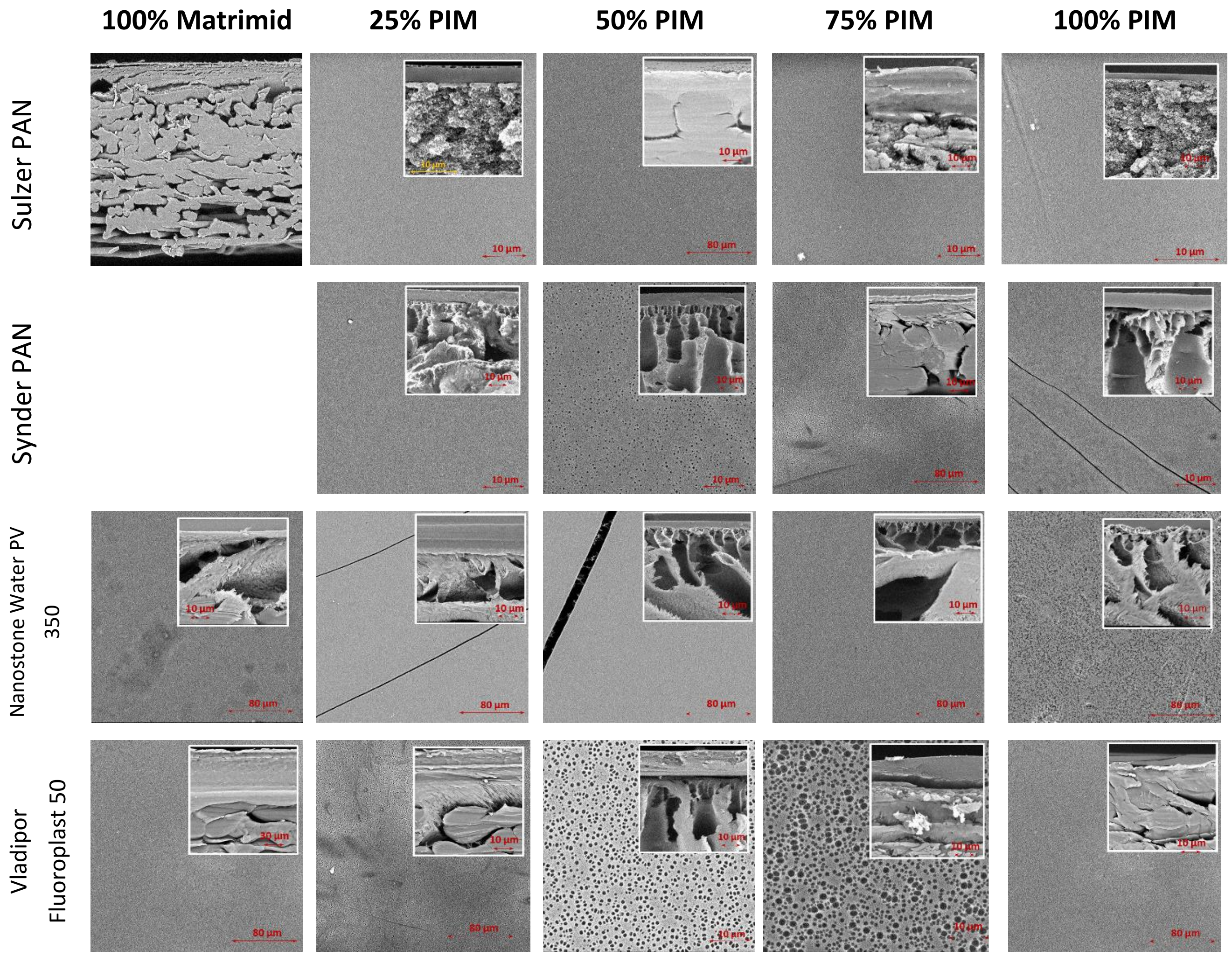

SI Figure S2. Cross-sectional SEM images of the of Matrimid®5218 / PIM-EA(Me2)-TB blend membranes with different blend compositions

		**Permeance [GPU]**					**Selectivity**			
SUPPORT	PIM-EA(Me$_2$)-TB [wt%]	N_2	O_2	CO_2	CH_4	H_2	CO_2/N_2	CO_2/CH_4	O_2/N_2	H_2/N_2
S1	0	0.1	0.3	1.6	0.1	4.8	25.7	26.3	5.18	75.2
	25	0.1	0.7	3.4	0.2	7.1	26.2	22.3	5.34	55.0
	50	0.3	1.5	6.9	0.3	16.5	23.1	22.6	4.97	54.7
	75	1.4	6.2	26.9	1.4	49.8	19.0	19.7	4.35	35.2
	100	4.9	32.7	129.0	6.5	402.7	26.1	19.7	6.62	81.6
S2	0	0.1	0.6	2.7	0.1	8.2	26.1	27.3	5.47	77.9
	25	0.2	0.9	3.9	0.2	10.7	21.7	18.8	4.82	59.5
	50	0.2	1.2	5.0	0.2	18.8	25.7	22.1	5.93	95.7
	75	0.3	1.3	4.9	0.3	26.0	18.8	16.8	4.89	100.4
	100	0.6	1.6	5.5	0.7	25.7	9.9	8.1	2.91	46.2
S3	0	2.7	2.8	4.7	3.8	13.3	1.8	1.3	1.05	4.9
	25	10.1	10.9	17.7	13.5	47.7	1.8	1.3	1.08	4.7
	50	98	96	119	131	374	1.2	0.9	0.98	3.8
	75	114	122	173	156	488	1.5	1.1	1.07	4.3
	100	441	419	430	584	1405	1.0	0.7	0.95	3.2
S4	0	389	356	333	540	1245	0.9	0.6	0.92	3.2
	25	71	66	71	100	262	1.0	0.7	0.92	3.7
	50	45	45	60	62	168	1.3	1.0	1.00	3.7
	75	1039	887	774	1099	1797	0.7	0.7	0.85	1.7
	100	-	-	-	-	-	-	-	-	-
